# Emergency Medical Service Utilization and Response Following COVID-19 Emergency and Stay-at-Home Policies: An Interrupted Time-Series Analysis

**DOI:** 10.7759/cureus.19794

**Published:** 2021-11-21

**Authors:** Allyson W O'Connor, Haylea A Hannah, Elisabeth A Burnor, Kai G Fukutaki, Troy Peterson, Dustin W Ballard, Rochelle R Ereman, Matthew D Willis, Orvalho J Augusto, Bradley H Wagenaar

**Affiliations:** 1 Health Services, University of Washington, Seattle, USA; 2 Epidemiology, University of Washington, Seattle, USA; 3 Department of Health & Human Services, County of Marin, San Rafael, USA; 4 Environmental and Occupational Health Sciences, University of Washington, Seattle, USA; 5 Keck School of Medicine of USC, University of Southern California, Los Angeles, USA; 6 Global Health, University of Washington, Seattle, USA; 7 Division of Research and San Rafael Medical Center, Kaiser Permanente, San Rafael, USA; 8 Faculdade de Medicina, Universidade Eduardo Mondlane, Maputo, MOZ

**Keywords:** emergency utilization, stay-at-home orders, public health policy, emergency medical services, covid-19 outbreak

## Abstract

Objective

Examine changing emergency medical services (EMS) utilization and response patterns associated with coronavirus disease 2019 (COVID-19) emergency declaration and stay-at-home orders during the first year of the COVID-19 pandemic.

Methods

We conducted an uncontrolled interrupted time series analysis of EMS calls (January 1, 2019 - March 1, 2021) in Marin County, California analyzing call volume (All calls, n=46,055); patient refusal of EMS care or transport and patient care resolved on scene (Calls with opportunity for transport; n=37,401); and call severity (Transported calls; n=27,887).

Results

Pre-COVID-19 (1/1/2019-3/2/2020), EMS transported patients were predominately female (50.6%), 80+ years old (31.6%), and Marin County residents (68.0%). During COVID-19 (3/3/2020-3/1/2021), EMS transported patients were predominately male (52.7%), 35-64 years old (29.8%), and Marin County residents (70.4%). After the first stay-at-home order on 3/17/2020, call volume immediately decreased by 48% (adjusted incidence rate ratio [aIRR]=0.52; 95% CI=0.35,0.79) for children (0-15 years) and 34% for adults 80+ years (aIRR=0.66;95% CI=0.46,0.95). The odds of a transported call being prioritized as severe doubled (adjusted odds ratio [aOR]=2.26; 95% CI=1.11,4.59). Though transport refusals increased by 69% for children after the first order (aOR, 1.69 [95% CI, 1.13-2.52]), immediately following the second order on 12/8/2020, transport refusals decreased by 30% for children but increased 38-40% for adults 35-79 years (aOR=1.40 [95% CI=1.04-1.89] for 35-64 years; 1.38 [95% CI=1.02-1.87] for 65-79 years). Calls resolved on scene by EMS increased after the first order among all ages and after the second order for adults 16-79 years.

Conclusions

Call volume reduced for children and older adults after the first COVID-19 stay-at-home order. Changes in call severity, patient care refusals, and on-scene care provided by EMS indicated a changing role for EMS during the outbreak.

## Introduction

Early in the coronavirus disease 2019 (COVID-19) pandemic, healthcare utilization declined across the United States [1]. COVID-19 is a deadly disease caused by the severe acute respiratory syndrome coronavirus 2 (SARS-CoV-2) virus, and alarm over community and hospital preparedness as the virus spread prompted public health authorities to prioritize COVID-19 care and treatment. Nonessential healthcare use was discouraged, and rationing of healthcare resources deliberately reduced healthcare utilization [2]. While most elective and nonessential medical procedures were deferred or cancelled [3], emergency care remained available to respond to urgent needs and the predicted influx of COVID-19 patients. 

Emergency medical services (EMS) are a vital source of pre-hospital medical care and transport, and reports of decreased EMS calls in the United States at the onset of the COVID-19 outbreak paralleled declines in emergency department (ED) use nationwide [2,4,5]. As the first cases of community-acquired COVID-19 were detected, public health policies were implemented to reduce disease spread. Emergency declarations mobilized resources, and stay-at-home orders were issued at state and local levels to limit person-to-person contact for all but essential activities [6]. In addition to rationing, reductions in healthcare use likely reflected lower demand for healthcare as people reduced travel and activity under stay-at-home orders. However, little is known about the effect of the COVID-19 outbreak on EMS practice patterns and patient interaction, particularly after emergency declarations and stay-at-home orders were implemented.

While perceptions of healthcare facilities as sources of contagion are common in infectious disease outbreaks [7], the decline in ED use at the start of the COVID-19 outbreak precipitated concerns that patients were foregoing or delaying necessary care with potentially adverse health effects [2,8]. EMS utilization and response patterns may serve as indicators of ED use and the acuity of patients arriving at ED facilities [1]. Information on the effect of outbreaks on EMS could aid public health and healthcare systems managing emergency care preparedness and resourcing [5,9]. The present study assessed changes in EMS call volume, care, and transport characteristics before (1/1/2019-3/2/2020) and after (3/3/2020-3/1/2021) local COVID-19 public health proclamations in the year after the outbreak began in one of the first US counties to issue a stay-at-home order - Marin County, California.

## Materials and methods

Study setting

Marin County, California issued a local emergency declaration in response to the COVID-19 outbreak on March 3, 2020, one week after the first regional case of community-acquired COVID-19 was confirmed. Marin County declared the first stay-at-home order in the US on March 17, 2020 in coordination with five surrounding counties [10]. This order extended through May 18, 2020 when phased re-opening of non-essential businesses occurred. A second stay-at-home order was issued on December 8, 2020 in response to increased COVID-19 hospitalizations and remained until phased reopening began on January 25, 2021. 

Marin County is located directly north of San Francisco and includes a mixture of suburban and rural communities. The county's estimated population of 258,826 people remained stable from 2019-2020 with nearly a third of residents aged 60 years or older [11,12]. Eleven EMS agencies serve three receiving hospitals in Marin County and transport approximately 15,000 patients annually [13]. While there were changes in EMS protocols specific to the COVID-19 outbreak, there were no changes to the type of EMS response criteria for release at scene [13]. 

Data source and outcomes 

The Marin County EMS agency electronically compiles patient care records from all EMS providers in Marin County. This study analyzed 46,055 de-identified records for calls made to EMS from January 1, 2019 through March 1, 2021. 

**Figure 1:**
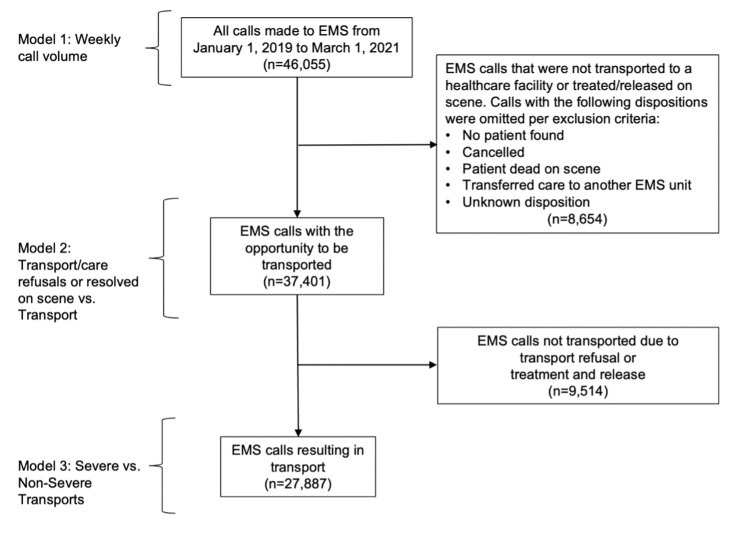
Diagram of Emergency Medical Service (EMS) calls included in each analysis - Marin County, California, January 1, 2019 to March 1, 2021 Model 1 examined trends in the weekly call volume of all EMS calls. Model 2 assessed the likelihood of a call resulting in a transport refusal or being resolved on scene by EMS as compared to transport to a healthcare facility. Model 3 evaluated the likelihood of a call being severe versus non-severe among calls transported by EMS.

Outcomes assessed in this study included (1) weekly EMS call volume, (2) EMS transport and care outcomes, and (3) patient severity (assigned to all patients transported by EMS). Figure 1 diagrams inclusion criteria of calls in each analysis. EMS call volume was measured as the weekly count of all EMS calls. EMS responders recorded patient disposition, or the outcome of a patient’s interaction with EMS, for each call. Patient disposition was used to identify calls included in transport, care, and severity analyses. 

Transport and care outcomes assessed the likelihood of patient refusal of transport or care and the likelihood of a patient being treated and released by EMS compared to being transported. For these analyses, calls to EMS were included if the patient (1) was transported by an EMS unit, (2) refused transport or care against medical advice, or (3) had care resolved on scene (treated and released, no treatment/transport required, or lift assists) by EMS (n=37,401). Calls were excluded from the transport and care analysis if (1) no patient was found on scene, (2) the call was cancelled prior to EMS arrival, (3) the patient was dead at the scene, regardless of on-scene resuscitation attempt, (4) the patient was transferred to the care of another EMS unit or after transport to a landing zone, or (5) patient disposition was unknown. 

In Marin County, EMS providers only assign a code indicating the severity level to calls resulting in transport to a healthcare facility (n=27,887). Severe transports were categorized as Code 3 calls requiring immediate transport with the use of lights and sirens. Non-severe transports were categorized as Code 2 calls that did not warrant lights and sirens.

Analytic plan 

The emergency declaration and stay-at-home order were identified a priori as important events influencing healthcare-seeking behaviors during the COVID-19 pandemic. Uncontrolled interrupted time series (ITS) analyses of EMS calls evaluated associations between these key events and EMS outcomes. 

We initially examined descriptive trends in EMS outcomes starting January 1, 2019 to inform ITS parameterization. Due to the weekly nature of the call volume analysis, trend periods began on the first Tuesday after each policy change. Our final models evaluated: (1) trends prior to the emergency declaration on March 3, 2020, (2) trends from the emergency declaration until the stay-at-home order (March 3-March 16, 2020), (3) immediate level changes after the stay-at-home order on March 17, 2020, (4) trends following the stay-at-home order (March 17-May 18, 2020), (5) immediate level changes after the second stay-at-home order on December 8, 2020, (6) trends following the stay-at-home order (December 8, 2020-January 25, 2021), and (7) trends following the second phased reopening (January 26-March 1, 2021). Trends and immediate level changes were interpreted as the comparative change from the previous period. 

Patient age was categorized as 0-15, 16-34 (reference category), 35-64, 65-79, or 80 years and older to capture medically relevant groupings. The distribution of patient characteristics (gender, age groupings, Marin County residency, and primary impressions) were examined pre- and post-emergency declaration to inform model development. Race and ethnicity were not evaluated due to missingness. Final models included annual sine and cosine seasonality terms given the seasonal nature of EMS call volume [14,15]. Given previous research indicating that EMS utilization varies by age, interactions between age group and each event parameter were also evaluated using likelihood ratio tests [16].

Weekly call volume (Model 1) was modeled using a multivariable generalized linear regression model with a negative binomial distribution. Transport and care characteristics (Model 2) were modeled using multinomial logistic regression with three outcome categories: (1) transported by EMS (reference category), (2) transport/care refusal, and (3) resolved on scene by EMS. Final call volume and transport/care models included interaction terms between age group and the immediate-level stay-at-home order parameter, as they significantly improved model fit. These models also included terms for time, event parameters, seasonality, and age group. 

Severity of transported calls (Model 3) was modeled using logistic regression. This model included time, event parameters, seasonality, and age group. Significant interaction was not observed between age group and any event parameter; thus, severity models did not include terms for interaction by age (all p>0.05). 

All analyses were performed in R 3.6.1 (R Foundation for Statistical Computing, Vienna, Austria) with the AER, MASS, sandwich, effects, and nnet packages and used two-sided hypothesis tests (α=0.05) [17-24]. The University of Washington Internal Review Board determined that this study was exempt from review for human subjects research.

## Results

Call characteristics

During the study period, 26,113 calls were made to EMS in the 15-month period prior to the COVID-19 emergency declaration in Marin County, and 19,942 calls were made to EMS in the year after the emergency declaration. Descriptive results of all EMS calls and calls resulting in transport by EMS are reported in Table 1.

**Table 1:** Characteristics of all Emergency Medical Service (EMS) calls (n=46,055) and EMS transported calls (n=27,887) – Marin County, California, January 1, 2019-March 1, 2021

	All EMS calls before emergency declaration (Jan. 1, 2019 – Mar. 2, 2020) n=26,113		All EMS calls after emergency declaration (Mar. 3, 2020 – Mar. 1, 2021) n=19,942		Transported EMS calls before emergency declaration (Jan. 1, 2019 – Mar. 2, 2020) n=16,138		Transported EMS calls after emergency declaration (Mar. 3, 2020 – Mar. 1, 2021) n=11,749	
Gender, No. (%)								
Female	10,924	(41.8)	7,928	(39.8)	8,165	(50.6)	5,517	(50.0)
Male	10,743	(41.1)	8,816	(44.2)	7,942	(49.2)	6,186	(52.7)
Missing	4,446	(17.0)	3,198	(16.0)	31	(0.2)	46	(0.4)
Age category in years, No. (%)								
0-15	925	(3.5)	547	(2.7)	535	(3.4)	280	(2.4)
16-34	2,678	(10.3)	2,185	(11.0)	1,711	(10.7)	1,305	(11.1)
35-64	6,261	(24.0)	5,004	(25.1)	4,460	(28.0)	3,507	(29.8)
65-79	5,341	(20.5)	4,424	(22.2)	4,200	(26.3)	3,327	(28.3)
80+	6,322	(24.2)	4,439	(22.3)	5,049	(31.6)	3,328	(28.3)
Missing	4,586	(17.6)	3,343	(16.8)	3	(0.02)	2	(0.02)
Marin County residency, No. (%)								
Resident	14,199	(54.0)	11,483	(57.6)	10,973	(68.0)	8,275	(70.4)
Non-resident	996	(3.8)	563	(2.8)	690	(4.3)	368	(3.1)
Missing	10,659	(41.8)	7,896	(39.6)	4,475	(27.7)	3,106	(26.4)
Top 10 primary impressions (%)								
1	Traumatic Injury (12.3)		Traumatic Injury (12.2)		Traumatic Injury (15.0)		Traumatic Injury (15.1)	
2	Pain (9.5)		Pain (8.9)		Pain (12.9)		Pain (12.3)	
3	Weakness (5.8)		Weakness (5.9)		Weakness (8.4)		Weakness (8.6)	
4	Altered Level of Consciousness (5.0)		Altered Level of Consciousness (4.4)		Altered Level of Consciousness (7.4)		Altered Level of Consciousness (6.7)	
5	Syncope (3.8)		Abdominal Pain/Problems (3.7)		Abdominal Pain/Problems (5.6)		Abdominal Pain/Problems (5.6)	
6	Abdominal Pain/Problems (3.7)		Syncope (3.3)		Syncope (4.9)		Respiratory Distress (5.0)	
7	Respiratory Distress (2.8)		Respiratory Distress (3.2)		Respiratory Distress (4.1)		Syncope/Near Syncope (4.1)	
8	No Apparent Illness/Injury (Adult) (2.6)		No Apparent Illness/Injury (Adult) (2.7)		Alcohol Intoxication (3.6)		Chest Pain – Suspected Cardiac (3.9)	
9	Alcohol Intoxication (2.6)		Chest Pain – Suspected Cardiac (2.4)		Chest Pain – Suspected Cardiac (3.3)		Alcohol Intoxication (3.1)	
10	Chest Pain – Suspected Cardiac (2.1)		Anxiety/Emotional Upset (2.3)		Nausea/Vomiting (2.4)		Nausea/Vomiting (2.3)	
Missing	6,167 (23.6)		4841 (24.3)		24 (0.1)		23 (0.2)	

Prior to the emergency declaration, calls transported by EMS were predominantly made for women (50.6%), those 80 years and older (31.6%), and Marin County residents (68.0%). After the emergency declaration, calls transported by EMS were predominantly made for men (52.7%), those 35 to 64 years old (29.8%), and Marin County residents (70.4%). The four most common primary impressions of patient health conditions were the same before and after the emergency declaration for all calls and transported calls: traumatic injury, pain, weakness, and altered level of consciousness. 

Call volume among all calls

**Figure 2:**
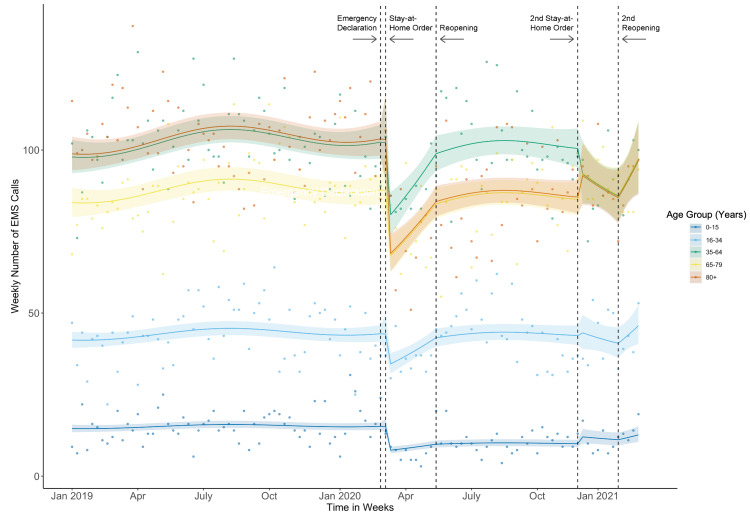
Weekly Emergency Medical Service (EMS) call volume and fitted model estimates stratified by age group – Marin County, California, January 1, 2019 to March 1, 2021 Model of fitted average call volume estimates and associated 95% confidence intervals plotted over crude point estimates of weekly EMS call volume by age group. Each fitted line represents the associated age group (see key) in years.

**Figure 3:**
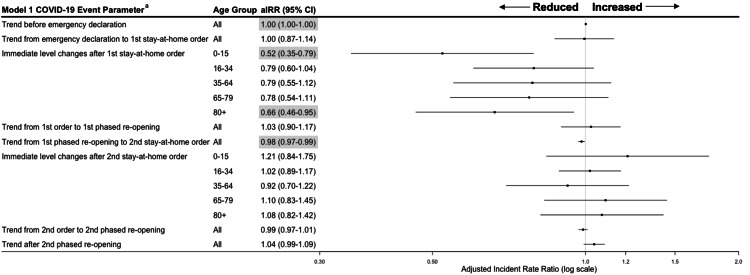
Weekly Emergency Medical Service (EMS) Call Volume adjusted incident rate ratios (aIRR) and 95% confidence intervals during COVID-19 outbreak periods – Marin County, California, January 1, 2019 to March 1, 2021 Incident rate ratios and 95% confidence interval highlighted in grey are statistically significant (p<0.05). ^a^Each trend parameter is compared to the trends from the period directly preceding in the incident rate ratio (e.g., the trend from emergency declaration to stay-at-home order compared to the trend pre-emergency declaration). Immediate-level changes compare the week directly before the stay-at-home order went into effect to the week immediately following the order’s implementation.

Weekly call volume trends were flat prior to the COVID-19 outbreak (Figure 2), and trends remained unchanged through the stay-at-home orders and re-openings (Figure 3). After the first stay-at-home order, there were immediate level decreases in call volume of 48% among children aged 0-15 years (adjusted incident rate ratio [aIRR], 0.52 [95% CI, 0.35-0.79]) and 34% among adults aged 80 years and older (aIRR, 0.66 [95% CI, 0.46-0.95]). Call volume also immediately decreased after the first stay-at-home order by 21-23% for adults aged 16-79 years (aIRR, 16-34 years: 0.79 [95% CI, 0.60-1.00]); 35-64 years: 0.79 [95% CI, 0.55-1.12]; 65-79 years: 0.77 [95% CI, 0.54-1.11]); however, these decreases were not statistically significant. Trends and immediate level effects on EMS call volume were not statistically significant following the first phased reopening, during, or after the second stay-at-home-order.

Transport and care among calls with opportunity for transport

**Figure 4:**
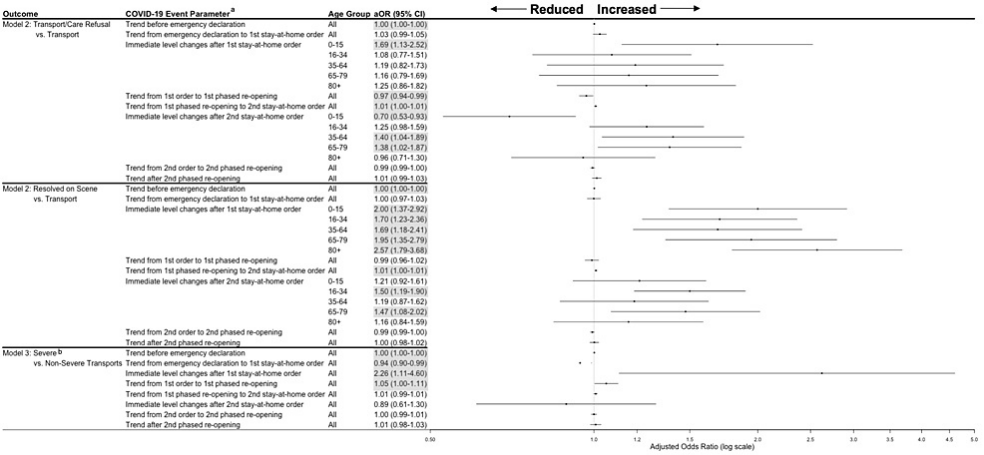
Transport and severity adjusted odds ratios (aOR) and 95% confidence intervals for Emergency Medical Service (EMS) calls during COVID-19 outbreak periods - Marin County, California, January 1, 2019 to March 1, 2021 Odds ratios and 95% confidence interval highlighted in grey are statistically significant (p<0.05). ^a^Each trend parameter is compared to the trends from the period directly preceding in the odds ratio (e.g., the trend from emergency declaration to stay-at-home order compared to the trend pre-emergency declaration). Immediate-level changes compare the day directly before the stay-at-home order went into effect to the day immediately following the order’s implementation. ^b^Statistically significant interaction was not observed between age group and any COVID-19 event parameter (p>0.05).

The adjusted daily trends in the odds of refusal were flat prior to the COVID-19 outbreak and after the emergency declaration (Figure 4). Following the first stay-at-home order, the daily trend in the odds of refusal decreased by 3% (adjusted odds ratio [aOR], 0.97 [95% CI, 0.94-0.99]), and the odds of transport refusals immediately increased by 69% for children 0-15 years old (aOR, 1.69 [95% CI, 1.13-2.52]). Trends in transport refusals were not statistically significant any time after the first phased reopening. However, immediately after the second stay-at-home-order, transport refusals decreased by 30% for children 0-15 years (aOR, 0.70 [95% CI, 0.53-0.93]) but increased by 38-40% for adults aged 35-79 years (aIRR, 35-64 years: 1.40 [95% CI, 1.04-1.89]; 65-79 years: 1.38 [95% CI, 1.02-1.87]).

Trends in calls resolved on scene were flat throughout the study period, though we observed immediate level increases in calls resolved on scene after both stay-at-home orders took effect (Figure 4). Following the first stay-at-home order, the immediate level change in the odds of a call being resolved on scene doubled for patients aged 0-15 years (aOR, 2.00 [95% CI, 1.37-2.92]), nearly doubled for patients aged 16-79 years (aOR 16-34 years: 1.70 [95% CI, 1.23-2.36]; 35-64 years: 1.69 [95% CI, 1.18-2.41]; and 65-79 years: 1.95 [95% CI, 1.35-2.79]), and increased 2.6-fold for patients 80 years or older (aOR, 2.57 [95% CI, 1.79-3.68]). Following the second stay-at-home order, calls being resolved on scene increased by 50% for adults aged 16-34 years (aOR, 1.50 [95% CI, 1.19-1.90]) and 47% for adults aged 65-79 years (aOR, 1.47 [95% CI, 1.08-2.02]).

Severity among transported calls

Pre-COVID-19 trends in the adjusted odds of a transported call being severe versus non-severe were flat (Figure 4). After the emergency declaration and before the first stay-at-home order, the trend in the odds of a severe transport decreased by 6% (aOR, 0.94 [95% CI, 0.90-0.99]). Immediately after the first stay-at-home order, the odds of a transported call being severe doubled (aOR, 2.26 [95% CI, 1.11-4.60]). Call severity increased 5% daily following the order until phased reopening began (aOR, 1.05 [95% CI, 1.00-1.11]). Trends and immediate level effects on the severity of transported calls were not statistically significant following the first phased reopening, during, or after the second stay-at-home-order.

## Discussion

Public health proclamations during the COVID-19 pandemic, like the emergency declaration and stay-at-home orders in Marin County, California, were definitive markers of change in EMS utilization, practice patterns, and patient behavior. The emergency declaration had little effect on EMS utilization, while the first stay-at-home order was a significant point of change for EMS. The first stay-at-home order was associated with increased call volume, on-scene care provided, transport refusals for children, and twice the likelihood of an EMS call being severe enough to require rapid transport. Immediately after the first order took effect, this study observed a 21-48% decline in call volume, depending on age group. These findings coincided with other observed declines in emergency care at the onset of the pandemic, including 26% decreases in EMS calls observed in two other US studies [5,25]. However, the present study observed statistically significant declines in call volume only for children 0-15 years old (48% decline) and adults aged 80 years and older (34% decline). This study expanded on descriptive reports of age-specific differences in EMS utilization throughout the pandemic by also evaluating how public health policies impacted this relationship.

Reduction in EMS call volume for children supports the expectation that a stay-at-home order limiting travel and activity would be associated with reduced injury or illness in children. Nationally, the largest proportional declines in ED visits during the pandemic were observed among those less than 15 years old [4]. It may also suggest changes in parental/guardian attitudes towards EMS transport as a means of limiting health system interaction. Children were the only age group with increased transport refusals after the first stay-at-home order, suggesting parents may have chosen to self-transport even after EMS contact was made. 

For adults aged 80 years and older, reduced call volume may corroborate initial concerns over patients foregoing care during the outbreak. While this study did not analyze whether call volume reduced due to delayed care, there was an observed two-fold increase in the severity of calls to EMS for all ages immediately after the first stay-at-home order. This result supports the theory that delays in seeking care may cause worsening of health conditions prior to EMS contact. However, this finding may also reflect reduced EMS use by patients with non-urgent conditions.

The decrease in EMS call volume and increase in patient severity observed after the first order were not observed after the second stay-at-home order. Furthermore, significant trends in call volume and severity were absent during and after the first phased reopening. Non-emergency or medically unnecessary calls to EMS for lower acuity patients tax EMS systems. Transports for individuals who lack alternative medical transportation [26] and up to one in six ambulance transports for children may be medically unnecessary [27]. A reduction in non-emergency calls to EMS potentially contributed to the reduced call volume observed immediately after the first stay-at-home order. If non-emergency calls remained low thereafter, e.g., out of continued hesitancy to interact with health systems, then further reductions in call volume following a second order would be attenuated. 

Following call volume and patient severity changes observed after the first stay-at-home order, there was additional evidence of ongoing changes in patient-EMS provider interactions. More care was provided by EMS on-scene for all age groups after the first stay-at-home order. After the second stay-at-home order, on-scene care increased for adults aged 16-79 years old. On-scene care did not change for adults 80 years and older as the conservative approach is often for EMS to immediately transport these patients. These findings highlight the changing role of EMS during a pandemic and are important to understand for EMS response and resourcing. 

Furthermore, while transport refusals were more likely only for children after the first order, this study found that adults contacting EMS were more likely to refuse care as the outbreak progressed. Transport refusals increased for adults aged 35-79 years but decreased for children after the second stay-at-home order. While rates of COVID-19 were very low in Marin County during the first stay-at-home phase, COVID-19 levels in the community increased over time. Increased care refusals by adults were potentially associated with fear of actual risk of contracting COVID-19 from the health system interaction more so than perceived risk early in the outbreak. 

EMS providers are an important source of pre-hospital care and maintaining the integrity and resilience of EMS during public health emergencies is paramount. Reductions in unnecessary calls and reduced demand due to changes in daily activity, e.g., lower risk of injury [6], after stay-at-home orders could take pressure off emergency care systems. More research is needed on the implications of EMS call volume, care, and transport on EMS resource allocation and for the acuity of patients presenting to EDs. EMS-attended medical emergencies receive faster hospital evaluation than those that use alternative modes of transportation [28]. Additional studies are needed to elucidate the nuanced complexities behind patient and EMS provider decision-making in the context of global pandemics.

Limitations

This study parameterized changes in EMS during the first year of the COVID-19 outbreak based on local public health emergency orders in one California county. However, associations observed in Marin County may provide insight for communities with similar policies. Additionally, other time-varying factors besides Marin County’s public health proclamations, such as media coverage of the pandemic, changing attitudes towards social distancing, hospital resourcing, and vaccine distribution, may have contributed to observed changes in EMS use and response.

Associations between the outbreak and EMS utilization could also vary by levels of COVID-19 circulating in the community and COVID-19 vaccinations. By the end of the first stay-at-home order on May 18, 2020, there were 348 COVID-19 cases, 41 COVID-19 hospitalizations and 14 deaths [29]. This low prevalence protected EMS outcomes against the influence of a COVID-19 patient surge during the first stay-at-home order. However, by the time of the second stay-at-home order, the community prevalence of COVID-19 was higher. By January 25, 2021, the end of the second stay-at-home order, there were 9,963 COVID-19 cases, 281 hospitalizations, and 156 deaths [29]. This difference in transmission levels paired with changing attitudes later in the pandemic may have limited the comparability of our findings between the first and second stay-at-home orders.

Missing age data in the call volume model was another limitation. Unlike the transport and severity models which included calls with greater patient-EMS interaction, the call volume model analyzed all calls made to EMS, regardless of whether EMS made contact. Age data were missing for nearly 20% of all calls, primarily due to the inclusion of calls cancelled prior to arrival or on scene. This potentially affected the age-adjusted associations in the call volume analysis. Lastly, the low average weekly number of calls observed among patients 0-15 years old may have limited study power and inference for this age group. 

## Conclusions

The most significant changes in EMS utilization and response occurred at the onset of the COVID-19 outbreak when little was known about virus transmission or prevalence of the disease and when health systems were least prepared. Assessment of multiple public health proclamations over the first year of the pandemic highlights the complex relationships between patient care-seeking behavior and EMS practice patterns. Though the first and second stay-at-home orders were similarly restrictive, EMS outcomes after the second stay-at-home order were unique. While EMS data may not be able to elucidate patient and responder attitudes and beliefs (e.g., fear or avoidance of seeking emergency care), it is a valuable tool for measuring EMS utilization and response patterns. This study also demonstrated the variation in EMS outcomes associated with COVID stay-at-home orders by age, information which could inform how emergency care systems may better serve their communities and offset care delays. Furthermore, results from this study underscore the need to examine health outcomes associated with emergency orders and rationing of care during pandemics. This knowledge may aid public health jurisdictions in building resilience into EMS and ED preparedness and resourcing during disease outbreaks.
